# Evolution of Liver Transplantation Indications: Expanding Horizons

**DOI:** 10.3390/medicina60030412

**Published:** 2024-02-28

**Authors:** Sara Battistella, Marco Grasso, Elisa Catanzaro, Francesca D’Arcangelo, Giorgia Corrà, Giacomo Germani, Marco Senzolo, Alberto Zanetto, Alberto Ferrarese, Martina Gambato, Patrizia Burra, Francesco Paolo Russo

**Affiliations:** Gastroenterology and Multivisceral Transplant Unit, Department of Surgery, Oncology and Gastroenterology, Padua University Hospital, 35128 Padua, Italy; sara.battistella.4@phd.unipd.it (S.B.); elisa.catanzaro95@gmail.com (E.C.); francesca.darcangelo02@gmail.com (F.D.); giorgia.corra@gmail.com (G.C.); germani.giacomo@gmail.com (G.G.); marcosenzolo@hotmail.com (M.S.); alberto.zanetto@unipd.it (A.Z.); alberto.ferrarese17@gmail.com (A.F.); martina.gambato@gmail.com (M.G.); burra@unipd.it (P.B.)

**Keywords:** liver transplantation, hepatocellular carcinoma, cholangiocarcinoma, acute alcoholic hepatitis, acute-on-chronic liver failure, colorectal liver metastasis, NET metastases

## Abstract

Liver transplantation (LT) has significantly transformed the prognosis of patients with end-stage liver disease and hepatocellular carcinoma (HCC). The traditional epidemiology of liver diseases has undergone a remarkable shift in indications for LT, marked by a decline in viral hepatitis and an increase in metabolic dysfunction-associated steatotic liver disease (MASLD), along with expanded indications for HCC. Recent advancements in surgical techniques, organ preservation and post-transplant patients’ management have opened new possibilities for LT. Conditions that were historically considered absolute contraindications have emerged as potential new indications, demonstrating promising results in terms of patient survival. While these expanding indications provide newfound hope, the ethical dilemma of organ scarcity persists. Addressing this requires careful consideration and international collaboration to ensure equitable access to LT. Multidisciplinary approaches and ongoing research efforts are crucial to navigate the evolving landscape of LT. This review aims to offer a current overview of the primary emerging indications for LT, focusing on acute-on-chronic liver failure (ACLF), acute alcoholic hepatitis (AH), intrahepatic and perihilar cholangiocarcinoma (i- and p-CCA), colorectal liver metastasis (CRLM), and neuroendocrine tumor (NET) liver metastases.

## 1. Introduction

Liver transplantation (LT) has drastically changed the natural prognosis of patients with end-stage liver disease (ESLD) and unresectable hepatocellular carcinoma under specific criteria. The 2019 annual data report from OPTN/SRTR confirmed a continual increase in waiting list (WL) registrations and performed transplants [1]. However, the COVID-19 pandemic caused a notable reversal of this trend in 2020, affecting both WL admissions and transplant activities, particularly in severely affected countries [1,2]. The surge in transplant activities has been coupled with substantial advancements in surgical techniques, patient selection, organ preservation, and anti-rejection therapies. Collectively, these improvements have resulted in enhanced post-transplant patient survival, with current 1- and 5-year overall survival rates at 86% and 74%, respectively [3]. Over the past decades, indications for LT have undergone a significant reevaluation due to changes in the epidemiology of liver diseases, marked by the introduction of effective antiviral drugs and shifts in lifestyle. Furthermore, LT is no longer solely viewed only as a curative measure but also as a strategy to improve survival compared to standard care. This conceptual shift poses a serious ethical challenge amid the organ shortage crisis, leading to unequal patient access to LT.

## 2. Viral Hepatitis and MASLD: The Changing Epidemiology

Hepatitis C virus (HCV) has traditionally been the predominant indication for LT in recent decades. However, the introduction of direct-acting antivirals (DAAs) has significantly alleviated the burden of HCV in transplant activities [4]. Flemming et al. analyzed the American SRTR database from 2003 to 2015, revealing a 30% reduction in WL admissions for HCV-related decompensated cirrhosis [5]. Similarly, Belli et al., using data from the European Liver Transplantation Registry (ELTR), observed a 58% decline in HCV as an indication for LT in decompensated cirrhosis and a 41% decrease for HCC [6]. This trend, particularly pronounced in patients with decompensated disease, was also confirmed in our center, where waiting list registrations for HCV-related ESLD decreased from 24.2% to 15.9% after the introduction of DAAs (*p* = 0.007) [7]. The role of DAAs in improving liver function was underscored by a high delisting rate after sustained virologic response (SVR). An analysis of 103 non-HCC HCV patients listed for LT showed a delisting rate of 34% following DAAs treatment for clinical improvement. DAAs also led to a notable improvement in post-transplant survival, rising from 65% to 77% at 3 years, virtually nullifying the risk of graft re-infection and its consequences [6].

Hepatitis B (HBV) continues to be a relatively common indication for LT in Western countries, and it stands as the leading cause in the Western Pacific region, Africa, Southeast Asia, and the Eastern Mediterranean Area [8,9]. Global vaccination campaigns and the introduction of nucleos(t)ide analogs (NUCs) have significantly altered the natural history of the disease. The primary goal of therapy with high-barrier NUCs is the long-term suppression of HBV DNA levels, with a functional cure being a rare event [10]; thus, the risk of HCC is reduced but not eliminated. Consequently, over the past 30 years, there has been a shift in indications for LT in HBV patients characterized by a reduction in transplants for decompensated disease and an increase in those performed for HCC [9].

Metabolic dysfunction-associated steatotic liver disease (MASLD) is currently the fastest-growing indication for LT in Europe, as well as particularly in the USA, where MASLD is now the second leading cause of LT [3,11]. This trend continues to rise for both decompensated cirrhosis and HCC, with an estimated increase of 168% and 137%, respectively, by 2030 [12]. It appears that MASLD patients more frequently develop HCC than non-MASLD individuals, with a significant proportion arising in non-cirrhotic livers [13,14].

## 3. Hepatocellular Carcinoma: Refining Selection Criteria

LT stands as the gold standard treatment for patients with early-stage non-resectable HCC, offering 5-year and 10-year survival rates of 70% and 50%, respectively, along with 5-year recurrence rates of 10–15% [15]. HCC has emerged as a prominent indication for LT, constituting over 25–30% of all transplants conducted in both the USA and Europe [3,16]. In Europe, the most common etiologies underlying HCC as indications for LT were HCV (43%), alcohol-related liver disease (27%), and HBV (16%) [3]. However, there has been a shifting landscape with an increasing incidence of MASLD in recent years [17]. This trend is even more pronounced in the USA, where MASLD has become the leading indication for LT in patients with HCC [18]. For many years, the Milan Criteria served as the cornerstone for selecting HCC patients. However, in the last two decades, the inclination to encompass patients with more advanced tumors and to consider not only the number and size of nodules but also biological markers has led to the formulation of new extended criteria. These criteria have demonstrated comparable survival and recurrence rates after LT (Table 1). In this context, the concept of downstaging has arisen, denoting the use of locoregional therapies (LRT) to diminish the tumor burden until it aligns with transplantability criteria. LRT is commonly employed to enhance the pool of potential candidates for LT [19]. However, data regarding downstaging treatments remain contentious due to several reasons, including variations in the types of LRT utilized, significant variability in the waiting period before LT across different centers, and notably the absence of intention-to-treat analyses [20]. UNOS/OPTN has standardized downstaging criteria, encompassing patients with a single lesion between 5 and 8 cm, 2–3 lesions ≤ 5 cm with the sum of maximum tumor diameters ≤ 8 cm, or 4–5 lesions ≤ 3 cm with the sum of maximum tumor diameters ≤ 8 cm, without vascular invasion or extrahepatic disease. Patients meeting these criteria can undergo LRT, and if they remain within the Milan Criteria for at least 3 months thereafter, they become eligible for LT. Modified RECIST criteria have been proposed to better evaluate treatment response in HCC patients, exhibiting a 2–3-fold improvement in the accuracy of the standard RECIST criteria and facilitating superior patient selection for LT [21,22]. Over the past few decades, liver allocation systems have undergone multiple revisions to prioritize patients with HCC, who frequently exhibit preserved liver function, on the waiting list for LT. However, despite the development of various scoring systems designed not only to forecast the risk of HCC recurrence post-LT but also to identify patients at a heightened risk of dropout from the waiting list, the current allocation prioritization treats all HCC patients uniformly, regardless of their liver dysfunction or tumor biology. Consequently, the current allocation scheme lacks precision in identifying patients at a higher risk of dropout [23].

Several post-transplant risk scores have been devised to predict the likelihood of recurrence after liver transplantation and guide post-transplant surveillance (Table 2). As of now, no adjuvant treatments are recommended to prevent HCC recurrence after LT. Portal vein tumor thrombosis is currently deemed a contraindication for LT in both European and American guidelines. However, recent studies have demonstrated acceptable results after LT in patients who underwent successful downstaging. Further evidence on patient selection and more precise trials are imperative for a comprehensive understanding of this aspect. Although numerous predictive models exist for assessing the risk of HCC recurrence both pre- and post-LT, there is currently a lack of specific guidelines to direct HCC surveillance following LT. Recent research by Lee et al. has shown that monitoring patients with imaging every 6 months during the initial 24 months post-LT is associated with the highest likelihood of detecting HCC recurrence at a stage amenable to aggressive post-recurrence treatment, leading to improved outcomes [24]. The RETREAT score has been proposed as a guiding framework for post-transplant surveillance, suggesting no surveillance for patients with a RETREAT score of 0 [25]. Patients with RETREAT scores of 1–3 are recommended to undergo surveillance every 6 months for the first 2 years post-transplant, while those with a score of 4 are advised to continue 6-month surveillance for 5 years. For individuals with a RETREAT score exceeding 5, a more frequent surveillance schedule is suggested, with imaging every 3–4 months during the initial 2 years post-LT, followed by 6-month intervals until the fifth year after transplantation.

Prospective studies involving larger populations are urgently warranted to tailor HCC surveillance strategies following LT to individual patients’ needs.

**Table 1:** Pre-transplant models for the prediction of HCC recurrence after LT.

Criteria	Description	Overall Survival	Recurrence
Milan Criteria [26]	<1 lesion < 5 cm or <3 lesions, each <3 cm No vascular invasion No extrahepatic metastasis	5-year survival: 85%	5-year recurrence-free survival: 92%
UCSF Criteria [27,28]	1 lesion < 6.5 or <3 lesions, each <4.5 cm A total tumor diameter < 8 cm No vascular invasion	1-year survival: 90% 5-year survival: 75.2%	5-year recurrence-free survival: 81%
Hangzhou Criteria [29]	Total tumor diameter < 8 cm or >8 cm if G1 and G2 aFP < 400	5-year survival: 70.7%	5-year recurrence-free survival: 62.4%
Up-to-Seven Criteria [30]	Total of the size of the largest lesion in cm + number of lesions = 7	5-year survival: 71.2%	
Extended Toronto Criteria [31]	No upper limit on size and number of lesions No extrahepatic metastasis No evidence of venous or biliary invasion No cancer-related symptoms All lesions beyond Milan Criteria must be biopsied to evaluate the differentiation (poor differentiation excluded from LT)	5-year survival: 70%	5-year recurrence-free survival: 66%
AFP Model [32]	Size of nodules (≤3 cm, between 3 and 6 cm, or ≥6 cm) Number of nodules (1–3 or ≥4) AFP serum levels (≤100, between 100 and 1000, or >1000 ng/mL)	5-year survival: Low risk (score < 2) = 69.9% High risk (score > 2) = 40.8%	5-year recurrence rate: Low risk (score < 2) = 13.4% High risk (score > 2) = 45.3%
Seoul criteria [33]	Tumor size (<3, 3–5, 5–6.5, and >6.5) Tumor number (1, 2–3, 4–5, and >5) aFP < 20, 20–200, 200–1000, and >1000 ng/ml	Score: 3–6 (transplantable); 3-year survival: 79%	Score: 3–6 (transplantable); 3-year recurrence-free survival: 87%
University of Padova selection criteria [34,35]	Any size and number of tumors No vascular invasion No extrahepatic metastasis No poorly differentiated tumor (grades III and IV)	5-year survival: 75%	5-year recurrence-free survival: 92%
Metro-Ticket 2.0 [36]	(1) aFP < 200 and sum of number + size ≤ 7 (2) aFP between 200 and 400 and sum of number + size ≤ 5 (3) aFP between 400 and 1000 and sum of number + size ≤ 4	5-year survival: 79.7% within criteria vs. 51.2% beyond criteria	5-year recurrence-free survival: 89.6% within criteria vs. 46.8% beyond criteria
TTV/aFP score [37]	TTV < 115 cm^3^ aFP < 400 ng/mL No vascular invasion No extrahepatic disease	74.6% at 4 years	68% at 4 years
HALT-HCC [38]	Hypotenuse between tumor number and largest tumor size ln-aFP MELD-Na	5-year survival: 82%	
MORAL pre-LT [39]	Largest tumor size aFP Pre-operative NLR		5-year RFS Low risk: 99% Medium risk: 70% High risk: 56% Very-high-risk group: 0% (18% at 1 year)
SMC criteria [40]	Tumor size ≤ 5 cm aFP level ≤ 400 ng/mL	5-year survival: 86.8% within criteria vs. 23.3% beyond criteria	5-year recurrence-free survival: 88.4% within criteria vs. 42.1% beyond criteria
TRAIN score [41]	aFP slope ≥ 15 ng/mL per month Radiological response to LRT based on mRECIST NLR ≥ 5 at LT Months on waiting list	5-year survival: 67.5% within criteria vs. 23.5% beyond criteria	5-year recurrence rate: 8.9% within criteria vs. 30% beyond criteria
aFP/TTD score [42]	Total tumor diameter ≤ 8 cm aFP ≤ 400 ng/mL		5-year recurrence-free survival: 74.4%

**Table 2:** Post-transplant models for the prediction of HCC recurrence after LT.

Score	Description	5-Year RFS
RETREAT [43]	Sum of the largest tumor diameter and number of tumors aFP Vascular invasion	5-year recurrence risk - 2.9% if RETREAT = 0 - 75% if RETREAT score ≥ 5
MORAL post-LT [39]	Tumor diameter Tumor number Vascular invasion Tumor differentiation	5-year RFS Low-risk group: 97% Medium-risk group: 75.1% High-risk group: 49.9% Very-high-risk group: 22%
Decaens et al. [44]	Tumor number Largest tumor diameter Vascular invasion Tumor differentiation	5-year recurrence risk: 14.5% if score < 4 and 51.5% if score ≥ 5

## 4. New Indications for Liver Transplantation

### 4.1. Acute-on-Chronic Liver Failure

Acute-on-chronic liver failure (ACLF) manifests as a clinical syndrome resulting from the acute decompensation (AD) of underlying chronic liver disease (CLD) associated with hepatic or extrahepatic organ failure [45]. It is characterized by complications such as jaundice, ascites, gastrointestinal hemorrhage, and an increased 90-day mortality rate of 14% [45,46,47]. Roughly 30% of patients experiencing AD will progress to a severe systemic inflammatory response leading to hepatic and/or extrahepatic organ failure, with a median 28-day mortality rate of 32.8% without LT [48]. According to EASL-CLIF criteria, ACLF is classified into four stages, each distinguished by the presence and number of organ failures, resulting in 41% and 78% mortality at 90 days for ACLF grade 1 and ACLF grade 3, respectively [49]. The management of ACLF revolves around identifying and addressing the triggering event and providing supportive medical therapy. LT emerges as a potential life-saving treatment for ACLF patients [45]. The recently published EASL guidelines on ACLF state that LT should be considered for patients with a severe presentation (ACLF grade 2 or 3) [45]. However, the current allocation system does not adequately assess the waiting list mortality risk for patients with severe ACLF [50]. Delaying LT in these patients is associated with higher mortality rates while waiting for LT or after LT [51]. Therefore, pilot programs to prioritize this patient group on the waiting list are recommended.

An example of a pilot program is the ACLFLT program in the UK, which proposes to include multidisciplinary selected patients with a super-urgent priority on the waiting list. This initiative aims to provide an expected survival rate of more than 60% for patients receiving organs in this category [45]. Existing models (MELD/MELD-Na and CPT) are designed to stratify patients with decompensated cirrhosis, overlooking the marked differences presented by ACLF [52]. The most accurate model for assessing short-term mortality risk in ACLF patients is the CLIF-C ACLF score, which incorporates the number of organ failures (calculated with CLIF-OF), age, and white blood cell (WBC) count, serving as a surrogate for systemic inflammation severity [49]. For ACLF grade 3 patients, the Transplantation for ACLF-3 Patients model (TAM score) has been developed to enhance the prediction of post-transplant survival [53,54,55]. This score includes easily accessible variables such as age (<53 or ≥53 years), blood lactate levels (<4 or ≥4 mmol/L), leukocytosis (≤10 or >10 g/L), and respiratory failure requiring pulmonary ventilation. Patients with TAM scores greater than 2 exhibit unfavorable post-transplant outcomes with a 1-year mortality rate of 84%. Pre-transplant blood lactate levels also appear predictive of post-transplant outcomes, as evidenced by the larger cohort of the ECLIS study [56]. Bacterial infections are the most common precipitating factors for the development of an ACLF, followed by alcohol abuse. Earlier data on the survival of patients undergoing LT for ACLF were not promising, with a 1-year post-LT survival rate of 75.3% in ACLF patients from the CANONIC study, lower than that observed in those undergoing LT for other indications [57]. However, subsequent studies reported high 90-day survival post-LT in patients with ACLF grades 1 and 2 and notably lower survival rates in those with grade 3 ACLF [58,59,60]. A multicenter study, including 250 patients, demonstrated a 1-year survival post-LT of 83.9% in ACLF 3, compared to only 7.9% in the non-LT control group [61].

Recent large-scale European investigations reported survival rates above 80% across all ACLF grades [56]. From various studies, risk factors associated with higher post-LT mortality include mechanical ventilation during respiratory failure, hemodynamic instability, uncontrolled sepsis, and more than four organ failures [56,61,62]. Therefore, carefully selecting patients with severe ACLF can yield substantial survival benefits.

### 4.2. Acute Alcoholic Hepatitis

Alcohol-related liver disease (ALD) stands out as one of the most prevalent forms of acute liver injury worldwide [63]. ALD encompasses a spectrum of liver insults resulting from alcohol abuse disorder (AUD), encompassing alcoholic hepatitis (AH), alcohol-associated cirrhosis (AC), and acute AH presenting as ACLF [64]. ALD remains a predominant and enduring indication for LT, contributing to half of liver-related deaths, with 10% of ALD-related deaths in the United States attributed to AH [65]. Among individuals with AUD, 35–40% will eventually develop AH, a clinical syndrome marked by acute liver decompensation due to extensive activation of liver inflammatory mechanisms following recent substantial alcohol consumption [66]. AH is associated with an overall mortality of 40–50%, but this can be as high as 70% in medical treatment-refractory patients [67,68,69,70]. Despite broad approval for LT in patients with AC, the role of LT in AH remains contentious. Given the high short-term mortality associated with AH, the early identification of patients who may benefit from LT is crucial. The Modified Maddrey’s Discriminant Function (mDF) was the initial prognostic score developed for AH, indicating patients requiring steroid therapy [64,71]. While mDF and the Model for End-Stage Liver Disease (MELD) were comparable, MELD’s ease of application in clinical practice makes it a preferred choice. Baseline MELD scores have been shown to predict death risk accurately in AH patients [72]. The Age, Bilirubin, INR, and Creatinine (ABIC) model independently predicts 90-day mortality and stratifies patients based on their risk of death at 90 days and one year [73,74]. The Glasgow Alcoholic Hepatitis Score (GAHS) showcases an impressive overall accuracy of 81% on day 1 when predicting a 28-day outcome [75]. Furthermore, its efficacy persists with similar results on days 6–9, predicting outcomes at 48 days when the score is applied within this timeframe. Within the validation cohort, the GAHS was more accurate than the mDF and MELD scores [75].

The Lille model, with a cutoff of 0.45 or less, offers high sensitivity and specificity for the early identification of patients at high risk of death, aiding in the assessment of corticosteroid response [76]. This model has demonstrated consistent accuracy when calculated at either day 4 or day 7 from the initiation of corticosteroid therapy [77]. An early evaluation at day 4 proves advantageous, enabling a prompt assessment of patients who may require LT [77].

In recent decades, patients with ALD faced a requirement of six months of abstinence before gaining access to transplantation programs. However, due to the notable six-month mortality in ALD patients and a lack of evidence supporting improved outcomes after LT, there has been a recent reevaluation of the six-month rule [78,79]. Early transplantation as a salvage therapy appears to be the most effective curative strategy [80]. Nevertheless, the scarcity of organ donations has brought forth ethical concerns regarding the perceived “self-inflicted” nature of alcohol-related disease.

**Figure 1:**
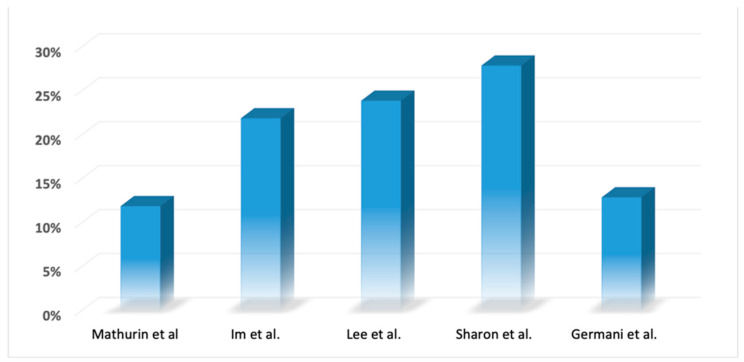
Reported recurrence of alcohol disorder after LT [81,82,86,87,88].

In 2011, a pivotal multicenter prospective study led by Mathurin’s group marked a turning point. This study demonstrated, for the first time, that LT in highly selected patients (Table 3) with severe AH could yield a significantly higher 6-month cumulative survival rate (77 ± 8% vs. 23 ± 8%, *p* < 0.001), with a low incidence of alcohol use after LT (only 3 out of 26 patients resumed drinking alcohol) [81]. This groundbreaking work laid the foundation for subsequent studies, such as the one by Germani et al., which reported a cumulative survival of 100% at 1 and 2 years in patients undergoing early LT for AH [82]. Inclusion criteria across these studies were generally consistent (Table 3), except for Sharon’s study, which included patients with a previous mental health disorder diagnosis, provided it was well managed. This divergence in criteria might explain the higher rate of alcohol use post-LT in Sharon’s study compared to other reports [82] (Figure 1). A comprehensive literature review involving 25 studies revealed that only 9 of them found the 6-month rule to be predictive of post-transplant abstinence [83]. Additionally, the “Group of Italian regions” within the Department of Health stated that the use of the 6-month rule is often applied without appropriate scientific evidence [84].

The recently published guidelines of the American College of Gastroenterology (ACG) state that, when patients with AH fail to respond to medical treatment, they may fulfill the “urgency, utility, and benefit” criteria commonly employed to select all eligible candidates for LT [85]. However, patient selection should not solely rely on the duration of sobriety but should also include a comprehensive evaluation of psychological and social support.

Numerous published studies have consistently shown that LT provides excellent survival benefits to patients with AH who undergo early transplantation following a rigorous selection process led by experienced teams. It is emphasized that alcoholism should be regarded as a disease, rather than a mere result of a bad habit. Consequently, it should no longer serve as a reason for excluding patients from optimal treatment perspectives, including LT.

**Table 3:** Studies on acute alcoholic hepatitis.

Study	Mathurin et al. [81]	Im et al. [86]	Lee et al. [87]	Sharon et al. [88]	Germani G. [82]
N°	26	9	17	46	16
Country or Region	Europe	United States	United States	United States	Europe
Comparison groups	Historic severe AH vs. no LT	Contemporaneous, severe AH and no LT	Contemporaneous, alcoholic cirrhosis with ≥ 6 months abstinence and underwent LT	Contemporaneous, alcoholic cirrhosis with ≥ 6 months abstinence and underwent LT	Severe AH vs. no LT
Inclusion criteria	First liver-decompensating event (i.e., no prior episode of alcoholic hepatitis or liver disease) Non-response to medical therapy (i.e., Lille Score > 0.45 or continuous increase in MELD) Presence of close supportive family members Absence of severe comorbid disorders Agreement to adhere to lifelong abstinence from alcohol Consensus about liver transplantation selection among a broad group of medical professionals	First liver-decompensating event Non-response to medical therapy (i.e., Lille Score > 0.45 after 7 days) Presence of good social support Favorable psychosocial profile, suggesting a low risk of alcohol relapse Agreement to lifelong alcohol abstinence	First liver-decompensating event Failure of medical management (i.e., Lille Score > 0.45 after 7 days, continuous increase in MELD) Commitment to lifelong adherence to alcohol abstinence (evaluated by an SA specialist) Strong social support from family and friends (evaluated by a transplant social worker) Rigorous assessment of possible risk factors for alcohol relapse by a substance abuse specialist and a social worker Absence of severe comorbid medical issues Full consensus agreement by a transplant committee	First liver decompensation with less than 6 months of sobriety Failure of medical management (i.e., Lille Score > 0.45 and worsening of MELD score) Patients were evaluated by a substance abuse specialist, as well as a social worker, to assess insight, commitment to abstinence, and the presence of strong social support Patients were required to be free of severe comorbid psychiatric or medical disease Patients with a previous diagnosis of mental health disorder, provided it was well managed	First liver-decompensating event Strong social support Absence of severe comorbid medical disorders Patient expected to adhere to lifelong alcohol abstinence
Survival 6-month 1-year 2-year	77% 71% NR	89% NR NR	100% 94% NR	98% NR NR	100% 100% 100%
Alcohol use post-LT (%)	12%	22%	24%	28%	12.5%
Median time to alcohol use post-LT (days)	740	132	83	256	174
Median Follow-up (days)	NR	730	548	532	1600

### 4.3. Intrahepatic and Perihilar Cholangiocarcinoma

#### 4.3.1. Intrahepatic Cholangiocarcinoma

Intrahepatic cholangiocarcinoma (iCCA) constitutes 10–20% of all cholangiocarcinoma cases, emerging as the second most common primary liver malignancy after HCC [89,90]. The predominant risk factors include infectious causes (liver flukes and viral hepatitis), biliary tract disease, metabolic syndrome, and cirrhosis. Intriguingly, more than 40% of cases present with no identifiable risk factor [91,92,93]. Despite therapeutic advancements, the incidence of iCCA has risen over the last decade, with only marginal improvements in overall survival [94]. Surgical resection remains the cornerstone of curative treatment for iCCA, but only about one-third of patients qualify for this option [94,95]. For those with unresectable iCCA, outcomes are bleak, yielding a median survival of 3–8 months, even with advancements in chemotherapy [96]. Moreover, resection often faces challenges due to a high recurrence rate. In pursuit of improved outcomes, LT has been explored as an option, particularly in cases of incidental findings of small iCCA (≤2 cm) on explant. Sapisochin et al. observed a remarkable 73% survival rate at 5 years for transplanted patients with such incidental findings [97]. An international multicenter study confirmed higher survival rates for “very early” iCCA (1-, 3-, and 5-year survival rates of 93%, 84%, and 65%, respectively), with a 5-year recurrence rate (18%) aligning with accepted standards for HCC outcomes [98]. These findings prompt a consideration of LT as a potential curative treatment for patients with “very early” iCCA. Recent research by De Martin and colleagues expanded the scope to patients with iCCA exceeding 2 cm but ≤ 5 cm, revealing lower recurrence rates (21% vs. 48%) and higher 5-year recurrence-free survival (74% vs. 40%) compared to resection [99]. This underscores the importance of patient selection beyond dimensional criteria, encompassing factors such as tumor biology and serum biomarkers. The role of neoadjuvant therapy before LT remains a subject of debate. While initial attempts were discouraging, recent pilot trials with gemcitabine plus platinum-based regimens have shown promise in downstaging locally advanced iCCA [100,101]. Combining LRT (stereotactic radiation or trans-arterial chemoembolization) with chemotherapy has yielded satisfying outcomes, yet a lack of strong evidence hampers clarity regarding the timing and selection of patients. In summary, LT for iCCA remains controversial and is not currently recommended outside of a trial setting [102]. There is a crucial need for concerted efforts to establish better selection criteria, thereby enhancing outcomes in the challenging landscape of iCCA treatment.

#### 4.3.2. Perihilar Cholangiocarcinoma

Perihilar cholangiocarcinoma (pCCA), situated in the anatomic area between second-degree bile ducts and the insertion of the cystic duct into the common bile tract, manifests as distinct in the extrahepatic and intrahepatic types [103]. Up to 10% of pCCA cases originate from underlying conditions such as primary sclerosing cholangitis (PSC), parasitic infections, intrahepatic lithiasis, choledochal cysts, or cirrhotic liver. Unfortunately, curative resection is viable in only 20–25% of patients with pCCA, and survival rates do not surpass 40% even in extensive cohort studies [104,105,106]. Adjuvant chemotherapy offers only a marginal improvement in survival [101]. LT, as a potentially curative treatment for unresectable pCCA, initially showed discouraging outcomes, with only 23% of patients surviving at 5 years and a recurrence rate exceeding 80% within 2 years post-transplantation [22]. However, advancements in chemotherapy have positively impacted clinical outcomes. The first evidence came from the association of radiotherapy–brachytherapy with chemotherapy [107,108]. The University of Nebraska pioneered a neo-adjuvant chemoradiation protocol involving intravenous 5-fluorouracil (5-FU) and brachytherapy before LT in a carefully selected patient group. This protocol achieved a recurrence-free survival of approximately 45% at a median follow-up time of 7.5 years [109]. Concurrently, the Mayo Clinic Center developed a protocol involving radio-sensitization through intravenous 5-FU injection, external beam irradiation, intraluminal brachytherapy, and the daily administration of capecitabine until LT. The protocol also included laparoscopic exploration before LT to exclude metastatic disease. The inclusion criteria comprised an established or highly suspected pCCA diagnosis (based on histology, brushing cytology, or the presence of malignant strictures at imaging associated with a carbohydrate antigen 19–9 (Ca19.9) higher than 100 ng/mL), unresectable disease < 3 cm, no evidence of intra- or extrahepatic metastasis, and medical suitability for LT [110]. Patients who had undergone a previous resection of the tumor prior to LT or percutaneous/surgical biopsy of the tumor were excluded [111]. The “Mayo Clinic Protocol” demonstrated a 5-year survival rate of 82% post-LT, comparable to LT for other chronic liver diseases and HCC [111]. A comparison between LT and resection revealed a 5-year survival rate of 82% vs. 21%, respectively, with fewer recurrences in the LT group [112]. Risk factors for disease recurrence post-LT included elevated Ca19.9, portal vein encasement, and the presence of residual tumor on the explant liver. Interestingly, underlying PSC was associated with better recurrence-free survival compared to the non-PSC group (72% vs. 51%) [110,111]. This gap might be potentially attributed to early cancer detection through surveillance and a better performance status at diagnosis [104].

An ongoing challenge is determining the optimal interval between neo-adjuvant therapy and LT. While a certain period before LT may aid in better patient selection, a prolonged staging-to-LT interval appears linked to increased recurrence rates [110]. The utilization of living donors could potentially address this issue and mitigate the impact of new transplant indications, such as unresectable pCCA, on WL [113]. Future clinical trials, such as the ongoing TRANSPHIL trial (NCT02232932), hold promise in providing additional insights into the potential applications of LT in settings beyond unresectable pCCA.

### 4.4. Colorectal Liver Metastases

**Figure 2:**
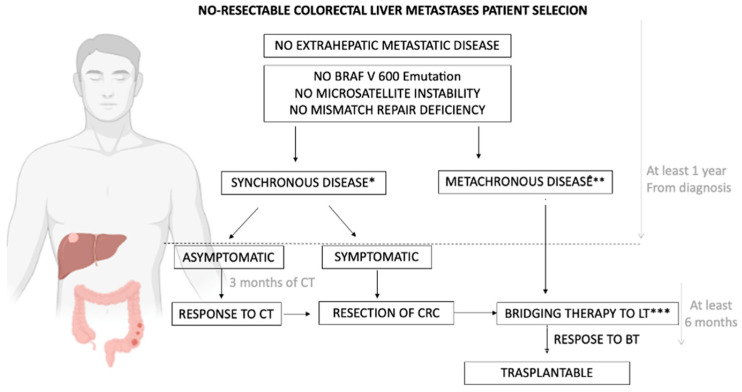
Proposed algorithm for the management of unresectable colorectal liver metastasis. * Synchronous disease: liver-only CRL metastases diagnosed (radiologically or histologically) before/at time of primary colorectal cancer. ** Metachronous disease: liver-only CRL metastases diagnosed within a year or after 1 year of primary colorectal cancer diagnosis. *** Bridging therapy: systemic therapy administered to evaluate biological behavior of liver metastases after primary tumor resection.

Colorectal cancer (CRC) is a significant global health concern, ranking as the third most prevalent malignancy and second most deadly. The incidence is expected to rise to 3.2 million new cases by 2040, with an increasing occurrence of early-onset CRC [114]. A substantial proportion (20–25%) of CRC cases are diagnosed at a metastatic stage, and up to half of these patients develop liver metastasis after primary tumor resection [115]. The sole curative approach for liver metastasis is resection, reporting a 5-year survival rate of 38% and a median survival of 3.6 years [115]. The concept of “oligometastasis” (OMD), defined as one to five treatable metastatic lesions in up to two sites with a controlled primary tumor, has opened avenues for potentially curative approaches, achieving long-term survival rates of 20–40% for patients with OMD confined to a limited number of sites [116]. While trials comparing surgical and non-surgical approaches for OMD are lacking, resection remains the standard treatment when feasible, although thermal ablation and radiotherapy have shown similar efficacy for small metastases [117,118]. For patients with CRC metastases not amenable to resection or LRT, chemotherapy (ChT) stands as the primary treatment option. According to recent ESMO guidelines, first-line treatment typically involves a ChT doublet (FOLFOX, FOLFIRI, and CAPOX) combined with anti-VEGF or anti-EGFR monoclonal antibodies (mAb) [119]. Despite continuous advancements in systemic therapies, the 5-year survival rate remains at 10% [120]. LT has been proposed for non-resectable liver metastases in CRC, raising ethical questions about organ allocation, patient selection, and WL management. Early attempts in 2002 by Starzl et al. were discontinued due to poor 5-year survival rates (18%) attributed to limited chemotherapeutic options at that time [121]. Over a decade later, the SECA-I trial, inspired by advancements in CRC treatments and a renewed interest in transplant oncology, showed promising outcomes. In this trial, 21 patients meeting strict criteria (good performance status, completely removed primary tumor, no other metastasis beyond the liver, and the completion of at least 6 weeks of ChT) underwent LT, achieving a 5-year survival rate of 60% [122]. However, the high recurrence rate (19/21)—although mostly treatable with locoregional therapies—raised questions about the need for a proper patient selection strategy and personalized immunosuppressive regiment after LT. The subsequent SECA-II trial, with more stringent patient selection criteria, achieved 5-year overall survival of 83% [123]. The Liver Transplantation for Colorectal Liver Metastasis 2021 working group, commissioned by the International Hepato-Pancreato-Biliary Association, proposed a patient selection algorithm (Figure 2) and emphasized the need for a personalized immunosuppressive regimen post-LT, minimizing calcineurin inhibitor exposure in favor of mTOR inhibitors [124].

Although liver graft scarcity and ethical concerns persist, LT for CRLM is an emerging field under investigation through various prospective trials, with promising results expected in the coming years (Table 4).

### 4.5. Neuroendocrine Tumors’ Liver Metastases

Neuroendocrine tumors (NETs) constitute a diverse group of neoplasms originating from neuroendocrine cells, exhibiting variability in localization, cellular differentiation, and endocrine secretory activity [125,126]. The small intestine (30%), appendix (20%), pancreas (16%), rectum (15%), and colon (13%) are common sites, whereas other localizations are less frequent [127]. Most NETs occur as sporadic tumors, but hereditary forms linked to genetic conditions, such as multiple endocrine neoplasia (MEN) 1 and 2, Von Hippel-Lindau syndrome, tuberous sclerosis complex, and neurofibromatosis type 1, may occur [128,129]. Metastases are present in 20–60% of patients at diagnosis, in particular for pancreatic NETs [130,131,132,133], with the liver being the primary site (up to 90% of cases) [134]. In the presence of distant metastases, the 5-year survival of pancreatic NET notably decreases from 80% to less than 30%, similar to what happens to other primary tumor localizations [127,135,136]. Surgical resection is the primary curative approach for metastatic NETs, offering a 5-year survival of up to 70% in patients with well-differentiated and focal metastases [137,138,139,140]. Unfortunately, only 15% of liver metastases are amenable to radical surgery [127,130]. Although not radical in patients with multifocal or bilobar metastasis, surgery might still improve their quality of life and survival, achieving better results than locoregional or systemic therapies [141,142,143]. For patients with unresectable metastases, LT has emerged as a valid alternative, although initial data were not highly promising. To optimize outcomes, the National Cancer Institute of Milan, led by the Mazzaferro group, introduced the “Milano Criteria” for selecting transplant candidates. These criteria include low-grade (G1–G2) NETs, metastases involving less than 50% of liver parenchyma, disease stability with a positive response to prior therapies for at least 6 months, and previous resection of the primary tumor with additional locoregional lymphadenectomy [134]. Primary tumor resection before LT is crucial, as outcomes are compromised if resection is performed during transplantation due to increased surgical risk, and only a few (but promising) data are available for the resection after LT. Interestingly, even in patients without an identifiable primary NET, LT has shown positive results, achieving a 5-year overall survival of 54% [144]. LT for NET liver metastasis is associated with an impressive 10-year survival probability of 88.8%, markedly higher than the 22.4% observed in non-transplant groups, and a significant reduction in disease progression to 13% [145]. The secretive activity of the primary tumor does not adversely affect survival, positioning LT as a strategy to control symptomatic disease [144]. A retrospective study comparing LT with resection in patients meeting Milan Criteria for NET metastases supported LT, demonstrating an improvement in 10-year survival from 75% to 93% and a substantial reduction in the 10-year recurrence rate from 18% to 52% [146].

In conclusion, for eligible patients with NET liver metastasis, surgical resection remains a potentially curative treatment with long-term survival [147]. LT is a consideration in strictly selected cases when resection is not feasible, and the use of marginal donors and living donors offers valuable options to address ethical concerns and minimize WL times. The evolving landscape of LT for pancreatic NETs necessitates ongoing research and the refinement of selection criteria to optimize outcomes in this challenging scenario.

## 5. How to Expand the Organ Pool

The increasing range of conditions warranting LT has led to a rise in the number of patients awaiting this procedure. As a result, there is a pressing need to expand the pool of potential liver donors. This has prompted the extension of acceptance criteria for liver grafts, including the incorporation of progressively extended criteria donor grafts, commonly known as marginal livers. These marginal livers encompass donations from older individuals, grafts with hepatic steatosis or those that are subjected to prolonged cold ischemia times, and donations after circulatory death (DCD). Nonetheless, the utilization of marginal grafts and DCD organs carries an increased risk of encountering primary non-function, early allograft dysfunction, and post-transplant cholangiopathy [148]. Currently, statically storing the graft at +4 °C represents the standard for liver preservation before transplantation. However, it is associated with ATP depletion, the accumulation of metabolites, and the gradual deterioration of cellular membrane functions. All these factors impose a stringent time limit on organ preservation.

Various strategies have emerged within the domain of dynamic perfusion: (1). Normothermic Regional Perfusion (NRP) [149], (2). ex situ Normothermic Machine Perfusion (NMP) [150], (3). Hypothermic Machine Perfusion (HMP) [151], and (4). sequential HOPE-NMP [152]. These approaches encompass diverse methods for sustaining and rejuvenating donor organs, ultimately augmenting their viability and potential for successful transplantation, thereby safely extending the donor pool [153].

Simultaneously, alternative approaches to broaden the donor pool, such as split liver transplantation and living-donor liver transplantation (LDLT), warrant exploration. LDLT is infrequently performed in Europe, accounting for only 5% of all transplant procedures [3]. This low utilization may be attributed to concerns regarding donor morbidity and mortality. However, in experienced centers, donor morbidity, specifically Clavien–Dindo complications ≥ 3, have been reported to be approximately 10% [154]. Moreover, LDLT circumvents restrictions imposed via the nationwide allocation system, as it involves private donation, [155] thus not affecting the deceased donor pool but, rather, benefiting all waitlisted patients [156].

## 6. Conclusions

The landscape of liver transplantation has undergone significant transformations in recent years, primarily driven by demographic shifts and advancements in etiological therapies. Notably, enhancements in both pre- and post-transplant management, as well as systemic oncological therapies, have broadened the scope of LT to encompass indications that were once deemed absolute contraindications. While this widening of transplant criteria signifies progress, it intersects with the enduring challenge of organ shortage, giving rise to substantial ethical dilemmas. The emphasis on strict candidate selection, multidisciplinary collaboration, and efforts to promote equitable access and allocation will be pivotal in navigating the ethical complexities associated with LT advancements.

## Figures and Tables

**Table 4:** Ongoing trials of liver transplantation for colorectal liver metastasis.

Trial Name (Number)	Location	Type of Study	Primary Endpoint	Actual Patients Enrolled	Study Period (Start–Estimated Completion Date)
TRANSMET (02597348)	France	Interventional, multicentric, parallel-assignment, randomized trial	5-year OS	94	February 2016–July 2026
LIVERT(W)O HEAL (03488953)	Germany	Interventional, bi-institutional, one-arm trial	OS 3 years after 2nd stage of hepatectomy	40 (estimated)	April 2018–December 2023
SECA III (03494946)	Norway	Interventional, randomized, parallel-assignment trial	OS 2 years after randomization	30 (estimated)	December 2016–January 2027
COLT (03803436)	Italy	Interventional, multicentric, non-randomized, open-label, controlled, prospective, parallel trial	5-year OS	22 (estimated)	January 2019– January 2024
TRASMETIR (04616495)	Spain	Multicentric, prospective, observational study cohort	5-year OS	30 (estimated)	September 2018–September 2028
MELODIC (04870870)	Italy	Multicentric, prospective, non-randomized, open-label, parallel trial	3- and 5-year OS	18 (estimated)	October 2020– October 2025
RAPID- PADUA (04865471)	Italy	Interventional, single-group assignment, clinical trial	Percentage of transplanted patients receiving second-stage hepatectomy within 4 weeks of segment 2/3 transplantation	18 (estimated)	October 2020–October 2025
EXCALIBUR 1 +2 (04898504)	Norway	Interventional, 3-arm, randomized, parallel-assignment trial	2-year OS	45 (estimated)	August 2021– May 2026
SOULMATE (04162092)	Sweden	Randomized, controlled, open-label, multicenter study	5-year OS	45 (estimated)	December 2020–June 2030

## Data Availability

No new data were created or analyzed in this study. Data sharing is not applicable to this article.
